# Recombinant Passenger Proteins Can Be Conveniently Purified by One-Step Affinity Chromatography

**DOI:** 10.1371/journal.pone.0143598

**Published:** 2015-12-07

**Authors:** Hua-zhen Wang, Zhi-zhan Chu, Chang-chao Chen, Ao-cheng Cao, Xin Tong, Can-bin Ouyang, Qi-hang Yuan, Mi-nan Wang, Zhong-kun Wu, Hai-hong Wang, Sheng-bin Wang

**Affiliations:** 1 College of Life Sciences, South China Agricultural University, Guangzhou, 541642, P. R. China; 2 Guangdong Provincial Key Laboratory of Protein Function and Regulation in Agricultural Organisms, Guangzhou, 541642, P. R. China; 3 Department of Pesticides, Institute of Plant Protection, Chinese Academy of Agricultural Sciences, Ministry of Agriculture, Beijing, 100193, China; 4 State Key Laboratory for Biology of Plant Diseases and Insect Pests, Beijing, 100193, China; Russian Academy of Sciences, Institute for Biological Instrumentation, RUSSIAN FEDERATION

## Abstract

Fusion tag is one of the best available tools to date for enhancement of the solubility or improvement of the expression level of recombinant proteins in *Escherichia coli*. Typically, two consecutive affinity purification steps are often necessitated for the purification of passenger proteins. As a fusion tag, acyl carrier protein (ACP) could greatly increase the soluble expression level of Glucokinase (GlcK), α-Amylase (Amy) and GFP. When fusion protein ACP-G2-GlcK-Histag and ACP-G2-Amy-Histag, in which a protease TEV recognition site was inserted between the fusion tag and passenger protein, were coexpressed with protease TEV respectively in *E*. *coli*, the efficient intracellular processing of fusion proteins was achieved. The resulting passenger protein GlcK-Histag and Amy-Histag accumulated predominantly in a soluble form, and could be conveniently purified by one-step Ni-chelating chromatography. However, the fusion protein ACP-GFP-Histag was processed incompletely by the protease TEV coexpressed *in vivo*, and a large portion of the resulting target protein GFP-Histag aggregated in insoluble form, indicating that the intracellular processing may affect the solubility of cleaved passenger protein. In this context, the soluble fusion protein ACP-GFP-Histag, contained in the supernatant of *E*. *coli* cell lysate, was directly subjected to cleavage *in vitro* by mixing it with the clarified cell lysate of *E*. *coli* overexpressing protease TEV. Consequently, the resulting target protein GFP-Histag could accumulate predominantly in a soluble form, and be purified conveniently by one-step Ni-chelating chromatography. The approaches presented here greatly simplify the purification process of passenger proteins, and eliminate the use of large amounts of pure site-specific proteases.

## Introduction

Although a number of expression hosts are available for the production of recombinant proteins, *Escherichia coli* expression system remains the first choice for basic research and the initial development in commercial activities owing to its several advantages, such as fast growth, low cost, and high-yield protein production [1]. About 90% of the structures presented in the Protein Data Bank were determined with the recombinant proteins produced in *E*. *coli* [2]. Up to 2011, 30% of the recombinant biopharmaceuticals licensed by the U.S. Food and Drug Administration (FDA) and European Medicines Agency (EMEA) have been produced in this host cell [3]. As a bacterial expression system, the limitation obviously exists for *E*. *coli* to express complex proteins due to lack of the sophisticated machinery to perform posttranslational modifications. Moreover, many recombinant proteins are liable to form incorrect conformation in this expression system, resulting in formation of inclusion bodies especially under over-expression condition. Up to 75% of human proteins are successfully expressed in *E*. *coli*, while only 25% of them are produced in an active soluble form using this host system [4].

Production of active soluble proteins continues to face challenges. To improve the solubility of recombinant proteins in *E*. *coli*, some factors have been intensively examined, such as culture temperature, combination of promoters and induction conditions, coexpression of molecular chaperones and folding modulators [1]. In some specific cases, enhancement of soluble protein production is significant. While, in other cases, improvements can be minimal and many recombinant proteins remain insoluble under these conditions. In light of the enhancement of soluble protein production, the best available tools to date have been fusion tags. Although the mechanisms for these fusion tags to enhance the solubility of their partner proteins remain unclear, several hypotheses have been proposed. It has been suggested that fusion partners can attract chaperones or have an intrinsic chaperone-like activities, and some acidic fusion partners can inhibit protein aggregation by electrostatic repulsion [5,6,7]. Parallel high throughput (HTP) screening using different fusion partners has greatly facilitated the rapid and cost-effective choice of the best fusion partner for a given target protein [2]. The repertoire of solubility-enhanced fusion tags has increased over years, with constant identification of novel fusion tags. Fusion tags are also commonly utilized for the production of toxic proteins, such as the expression of recombinant antimicrobial peptides (AMPs) in *E*. *coli* [8].

Using fusion tags to increase the soluble expression level or quench the toxicity of recombinant proteins is only the first step in the pathway towards target protein production. Many of fusion tags are large proteins, and may potentially interfere with the proper structure and function of passenger proteins. Biochemical investigations, especially production of recombinant proteins as pharmaceuticals require removal of these fusion tags. Both chemical and enzymatic methods have been used to cleave the fusion proteins for this purpose. Compared with enzymatic strategy, chemical cleavage offers a less expensive alternative, while it is less specific and often presents harsh conditions that can affect the stability or solubility of target proteins, and may cause side-chain modifications [9]. Furthermore, the frequent presence of more than one residue site recognized by those chemical reagents within the sequence of the target protein limits its use. The natural site-specific enzymes, such as tobacco etch virus protease (TEV), Enterokinase, Factor Xa, have been broadly used for removal of the fusion tags due to their requisite specificity and mild reaction condition [10]. For a given protease, the efficiency of enzymatic cleavage of the fusion proteins may vary with different proteins. Steric hindrance or the presence of unfavorable residues around the cleavage site applies to this inefficient processing. In the fusion protein MBP-SycH, the close proximity of the protease TEV recognition site to the structured N-terminus of SycH resulted in its failure to be cleaved by protease TEV, while the addition of five glycine residues between the recognition site and the N-terminus of SycH alleviated this impediment [11].

Removal of a fusion tag is usually accomplished by two consecutive affinity purification steps. Commonly, a histidine tag is fused with a fusion protein at its N-terminal. After being expressed, a His-tagged fusion protein is usually first purified via the initial affinity purification step, and then subjected to action of the His-tagged endoprotease to cleave off the fusion tag. Lastly, the target protein is recovered in the flow-through sample after a second affinity purification step, in which the cleaved-off fusion tag and the added endoprotease are immobilized on a matrix [12].

The purified site-specific proteases for tag removal usually are high expensive. For large scale production of the target proteins, which need to be expressed in fusion form, the expense related to use of the endoprotease accounts for a large portion of the manufacturing cost [9]. Otherwise, for production of the purified target proteins expressed in fusion form, two affinity purification steps are usually necessitated for separation of the fusion proteins and then removal of the fusion tags as well as endoproteases [12]. This two-step purification procedure is not only a tedious physical work, time-costing, and often results in the lower target protein productivity in light of the protein loss during purification. Development of a simplified procedure for purification of target proteins, which need to be expressed in fusion form, is of practical significance. In this report, we present two approaches for convenient purification of the target proteins expressed in fusion form, by which they can be separated with just one-step affinity chromatography. The approaches presented here can also eliminate the need of large amounts of pure endoproteases.

## Results

### Fusion expression and purification of alpha-Amylase and Glucokinase

**Fig 1:**
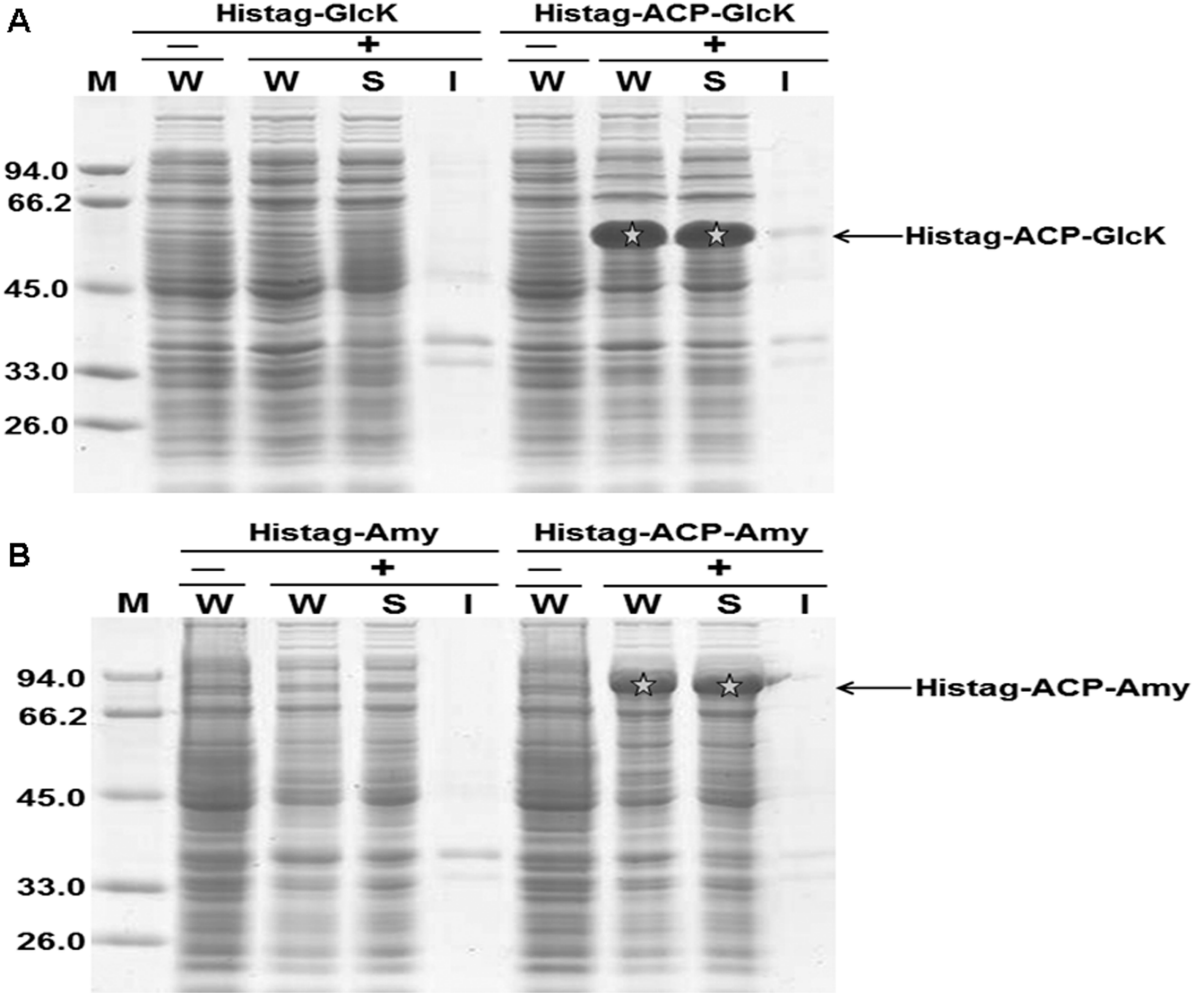
SDS-PAGE analysis of the expression of fusion protein ACP-GlcK (panel A) and ACP-Amy (panel B) in *E*. *coli* strain BL21 (DE3). After induction by addition of IPTG to 0.4 mM in the medium at 20°C for 12 h, *E*. *coli* cells were harvested. After sonication, the cell lysate was centrifuged to remove cell debris, then the supernatants were recovered and the insoluble fraction was assumed to consist of inclusion bodies. W: whole cell lysate; S: supernatant; I: inclusion bodies; +: IPTG was added to culture medium to induce the expression of recombinant proteins; -: no IPTG was added.

α-Amylase (Amy) is one of the most widely used technological enzymes, with broad applications in food processing and other industries that use starch liquefaction [13,14]. Glucokinase (GlcK) plays an important role in glucose homeostasis. In the liver, phosphorylation of glucose by GlcK promotes glycogen synthesis, while in the pancreatic β-cell its activity brings about insulin release [15]. Glucokinase and α-Amylase gene form *Bacillus subtilis* were cloned to the expression vector pET28b respectively, and then induced to express in *E*. *coli* by addition of IPTG to the medium. The expression levels of these two proteins were much lower than expected. Heterogenic expression of the recombinant proteins in *E*. *coli* often meets many obstacles, such as insolubility, instability and so on. To solve these problems, fusion tags have been widely used [10,16]. Acyl carrier protein (ACP) is a small acid protein, with a molecular weight of 8.5 kDa [17]. To increase the expression levels of these proteins in *E*. *coli*, we tried to fuse GlcK and Amy to the C-terminus of ACP respectively. As expected, the expression levels of these recombinant proteins were increased significantly, and a large portion of them accumulated predominantly in a soluble form (Fig 1).

**Fig 2:**
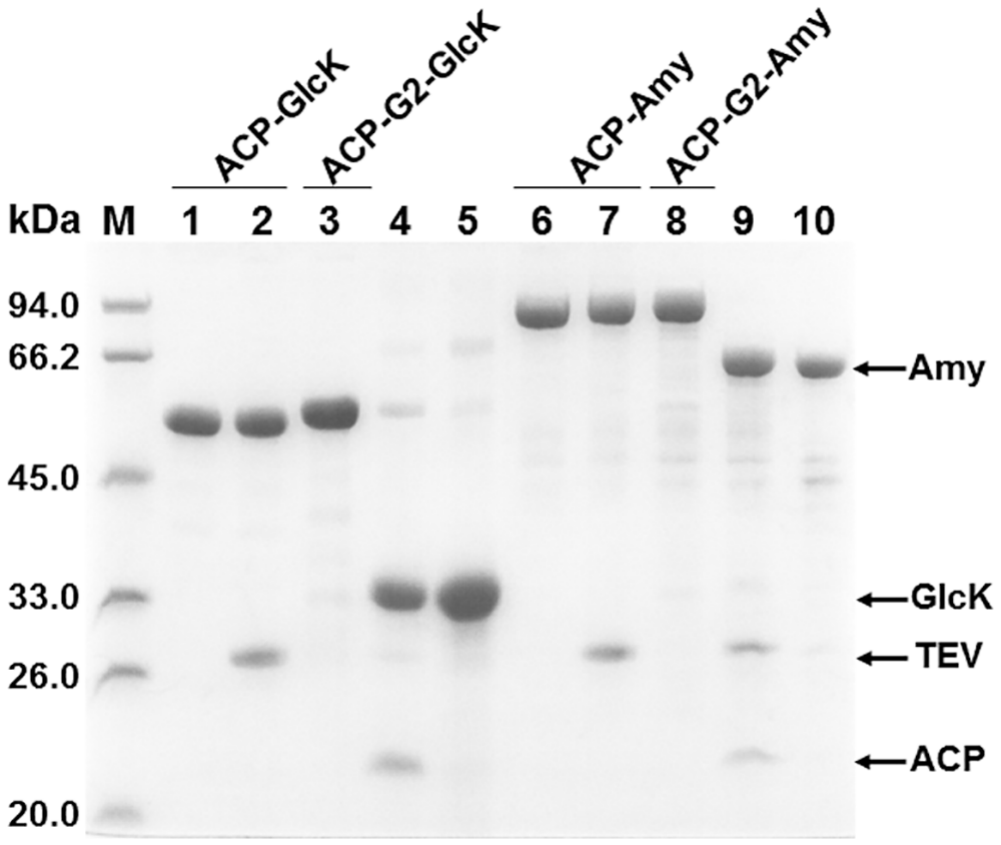
Purification of the passenger proteins (target proteins) by two consecutive affinity chromatography steps. Recombinant fusion proteins were first purified by Ni-chelating affinity chromatography. After that, the fusion proteins were subjected to processing *in vitro* by mixing them with the purified His-tagged protease TEV. Lastly, the resulting target proteins were separated by additional Ni-chelating affinity chromatography to remove His-tagged protease TEV and the fusion tag ACP. All samples were subjected to 12% SDS-PAGE analysis, lane 1, purified fusion protein Histag-ACP-GlcK; lane 2, the purified fusion protein Histag-ACP-GlcK cleaved *in vitro* by pure His-tagged protease TEV; lane 3, purified fusion protein Histag-ACP-G_2_-GlcK; lane 4, the purified fusion protein Histag-ACP-G_2_-GlcK cleaved *in vitro* by pure His-tagged protease TEV; lane 5, purified target protein GlcK; lane 6, purified fusion protein Histag-ACP-Amy; lane 7, the purified fusion protein Histag-ACP-Amy cleaved *in vitro* by pure His-tagged protease TEV; lane 8, purified fusion protein Histag-ACP-G_2_-Amy; lane 9, the purified fusion protein Histag-ACP-G_2_-Amy cleaved *in vitro* by pure His-tagged protease TEV; lane 10, purified target protein Amy.

Routinely, passenger protein (target protein) Amy and GlcK could be purified according to the two-step affinity purification procedure reported [12]. Recombinant fusion protein Histag-ACP-Amy and Histag-ACP-GlcK were purified by Ni-chelating affinity chromatography, and then subjected to processing *in vitro* by addition of the site-specific protease TEV to cleave off the fusion tag Histag-ACP. Unexpectedly, these fusion proteins were all refractory to the action of protease TEV. The structured protein domains flanking the TEV recognition site (ENLYFQ) in fusion proteins probably affect the interaction between protease TEV and its recognition site due to the steric hindrance. We reconstructed the fusion protein Histag-ACP-G2-Amy and Histag-ACP-G2-GlcK. Compared with their corresponding counterparts, two consecutive glycine residues were inserted between the protease TEV recognition site and the N-terminus of passenger protein. As expected, fusion protein Histag-ACP-G2-Amy and Histag-ACP-G2-GlcK were all ready to be cleaved *in vitro* by the purified His-tagged protease TEV. After second subtractive Ni-chelating affinity chromatography to remove the His-tagged ACP and His-tagged protease TEV, target protein Amy and GlcK were all eventually purified to high homogeneity (Fig 2).

### Intracellular processing of the fusion proteins by coexpression of the protease TEV

Generally, target proteins expressed in fusion form can be purified by two-step affinity chromatography, with separation of the fusion proteins and subsequent removal of the fusion tags as well as site-specific protease [10]. However, compared with the commonly used procedure for purification of the recombinant proteins by one-step affinity chromatography, this procedure often necessitates additional desalination of protein samples and the second affinity chromatography, leading to a larger possible loss of target proteins.

**Fig 3:**
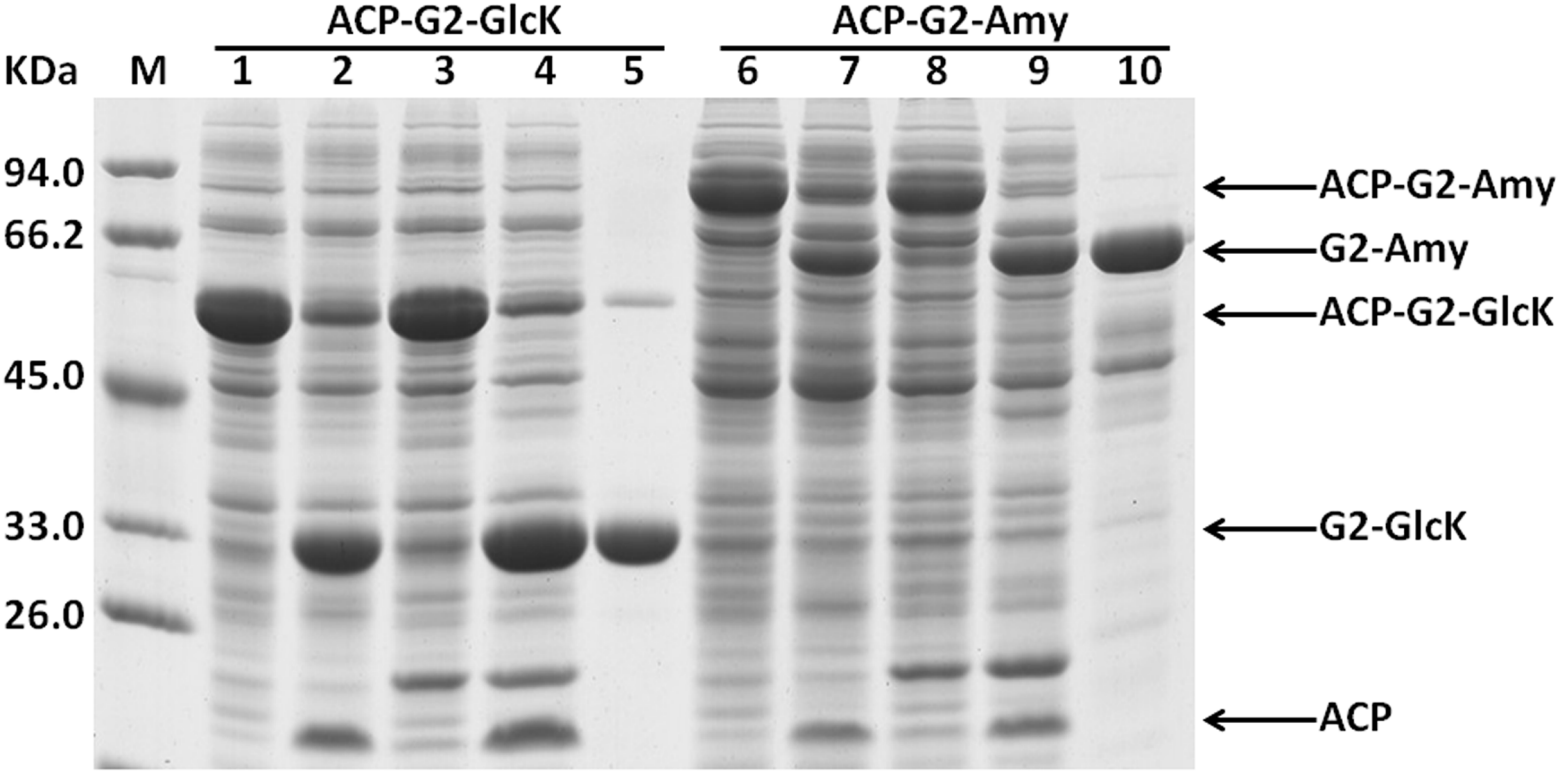
Intracellular processing of fusion protein ACP-G2-GlcK-Histag and ACP-G2-Amy-Histag by the protease TEV coexpressed in *E*. *coli* and subsequently purification of the target protein GlcK and Amy by one-step Ni-chelating chromatography. The clarified supernatants of *E*. *coli* cell lysate and purified proteins were subjected to 12% SDS-PAGE analysis, lane 1 and lane 6, the supernatant of the cell lysate of *E*. *coli* expressing fusion proteins; lane 2 and lane 7, the supernatant of the cell lysate of *E*. *coli* expressing fusion proteins treated by addition of the purified protease TEV; lane 3 and lane 8, the supernatant of the cell lysate of *E*. *coli* coexpressing the fusion proteins with protease TEV, which was expressed by vector pBAD34-MBP-TEV-WHZ; lane 4 and lane 9, the supernatant of the cell lysate of *E*. *coli* coexpressing the fusion proteins with protease TEV, which was expressed by vector pSU18-MBP-TEV-WHZ; lane 5 and lane 10, purified target proteins.

When a protease TEV recognition site was inserted between the fusion tag MBP and protease TEV, the recombinant fusion protein MBP-TEV expressed in *E*. *coli* could be efficiently self-cleaved *in vivo* [18,19]. These results imply that recombinant protease TEV can exhibit high activity, and may efficiently cleave the co-existing substrates *in vivo*. These accumulated data promoted us to investigate intracellular cleavage of the recombinant fusion proteins by the protease TEV coexpressed. Expression vector pSU18-MBP-TEV and pBAD34-MBP-TEV were constructed respectively for expression of the protease TEV. We first co-transformed the vector pET-28b-ACP-G2-GlcK-Histag with pSU18-MBP-TEV to *E*. *coli*. As shown in Fig 3, recombinant fusion protein ACP-G2-GlcK-Histag could be efficiently cleaved *in vivo* by the protease TEV coexpressed, leading to the accumulation of soluble His-tagged target protein GlcK-Histag. By just one-step Ni-chelating affinity chromatography, recombinant target protein GlcK could be conveniently separated. However, when vector pET-28b-ACP-G2-GlcK-Histag and pBAD34-MBP-TEV were co-transformed to *E coli*, recombinant target protein GlcK expressed *in vivo* remained in fusion form, suggesting that the fusion protein ACP-G2-GlcK-Histag did not undergo efficient cleavage *in vivo*. In fact, for undetermined reasons, the expression of protease TEV in *E*. *coli* could hardly been detected with the vector pBAD34-MBP-TEV (data not shown). Similar results were also obtained with the fusion protein ACP-G2-Amy-Histag (Fig 3).

Overall, under coexpression condition, protein GlcK and Amy not only were successfully expressed in a soluble form with desired expression levels, they also could be separated conveniently by one-step affinity chromatography. The specific activity of recombinant Amy reached 580.36U (mg protein)^-1^ and 1.92U (mg protein)^-1^ respectively. Our data indicated that recombinant Amy and GlcK retained their natural activity.

**Fig 4:**
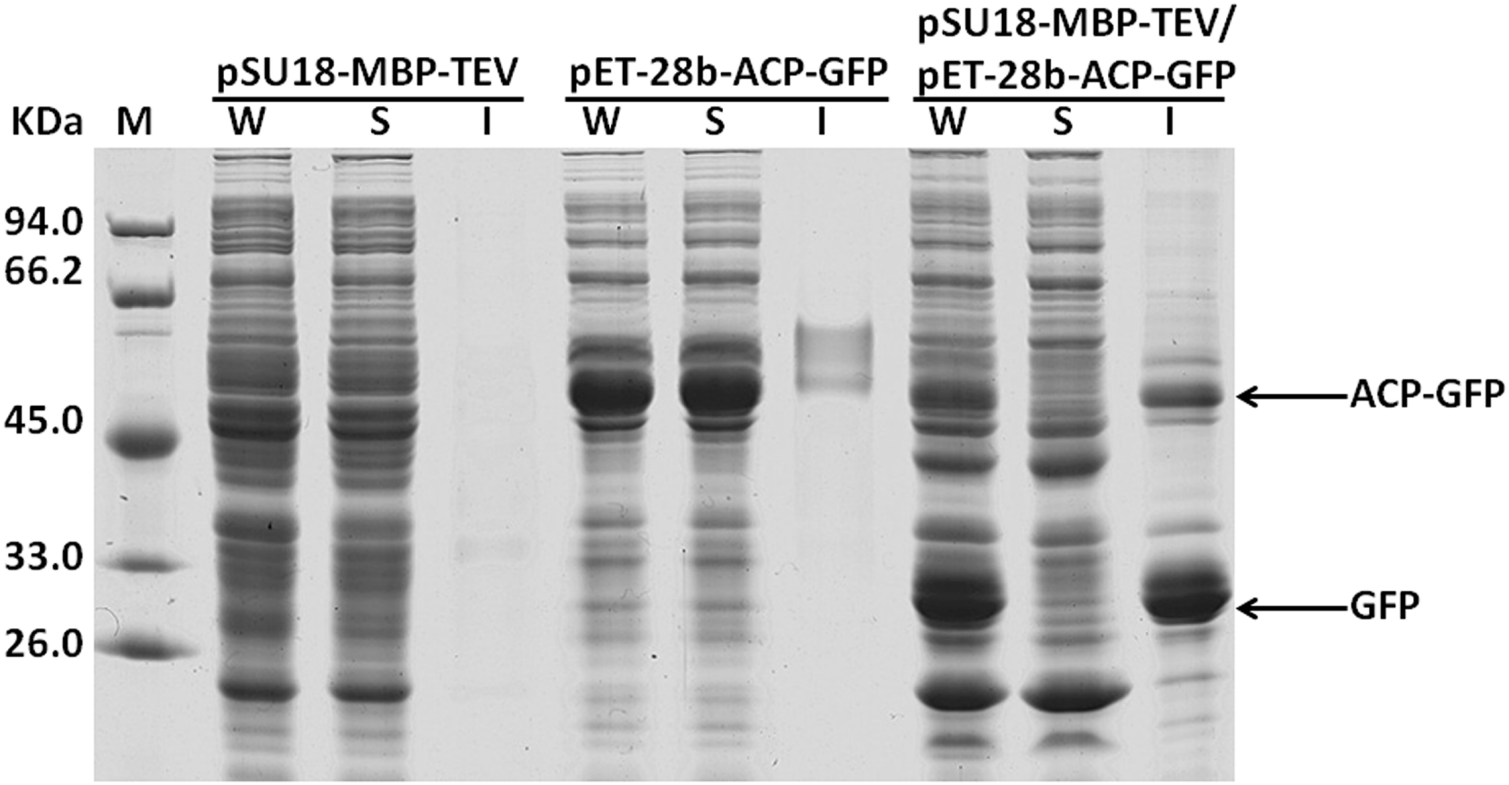
Intracellular processing of the fusion protein ACP-GFP-Histag by the protease TEV coexpressed in *E*. *coli*. *E*. *coli* cells were transformed with vector pSU18-MBP-TEV-WHZ and pET-28b-ACP-GFP-Histag respectively, or co-transformed with vector pSU18-MBP-TEV-WHZ and pET-28b-ACP-GFP-Histag. After induced by addition of IPTG to 0.4 mM in the medium for 12 h at 20°C, the *E*. *coli* cells were lysed by sonication on ice. After that, the cell lysate was centrifuged to remove cell debris, then the supernatants were recovered and the insoluble fraction was assumed to consist of inclusion bodies. W: whole cell lysate; S: supernatant; I: inclusion bodies.

Green fluorescent protein (GFP) tends to form inclusion bodies in *E*.*coli* when over-expressed in unfused form. As a fusion tag, ACP could also greatly enhance the solubility of recombinant GFP (data not shown). In this case, we constructed vector pET-28b-ACP-GFP-Histag, and investigated coexpression of the fusion protein ACP-GFP-Histag with protease TEV. Expression vector pET-28b-ACP-GFP-Histag was co-transformed to *E*. *coli* with pSU18-MBP-TEV, and then the expression of recombinant fusion protein ACP-GFP-Histag was analyzed. As shown in Fig 4, a portion of the fusion protein ACP-GFP-Histag could be cleaved *in vivo* by the protease TEV coexpressed, leading to the generation of target protein GFP-Histag. Nevertheless, the intracellular cleavage of fusion protein ACP-GFP-Histag was incomplete, and the resulting target protein GFP-Histag accumulated predominantly in an insoluble form.

### Development of a procedure for the convenient purification of passenger proteins via processing of the fusion proteins *in vitro*

**Fig 5:**
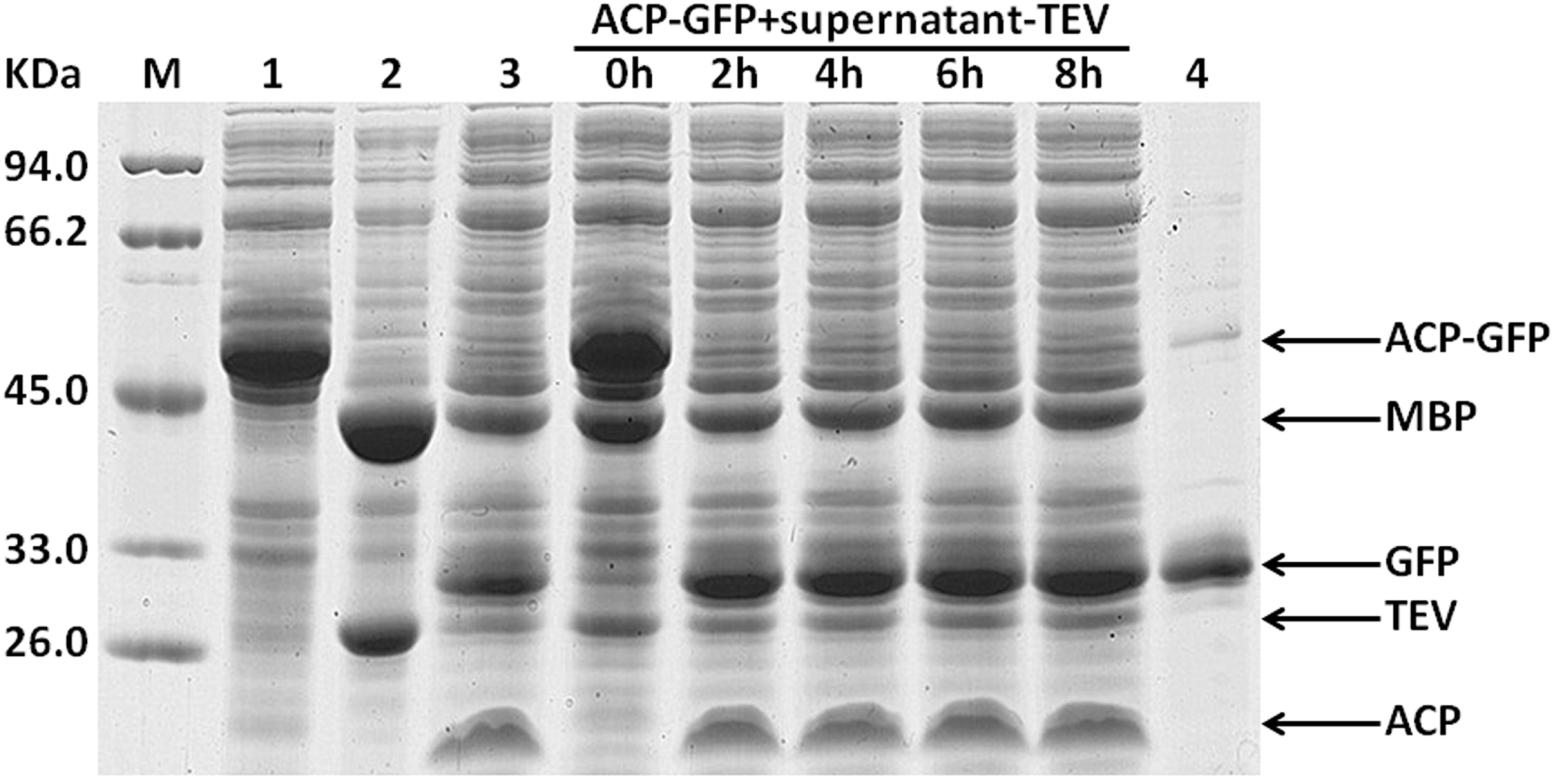
*In vitro* processing of the recombinant fusion protein ACP-GFP contained in *E*. *coli* cell lysate was performed by mixing it with the clarified cell lysate of *E*. *coli* over-expressing protease TEV (supernatant-TEV), and subsequently target protein GFP was purified of by one-step Ni-chelating chromatography. The supernatant of the cell lysate of *E*. *coli* expressing fusion protein ACP-GFP was mixed with supernatant-TEV at a volumetric ration of 10:1 and incubated at 25°C. Aliquots were removed for SDS-PAGE analysis after 0 h, 2 h, 4 h, 6 h and 8 h. lane 1, the supernatant of the cell lysate of *E*. *coli* expressing fusion protein ACP-GFP; lane 2, the supernatant of the cell lysate of *E*. *coli* expressing protease TEV by vector pET-28b-MBP-TEV-WHZ; lane 3, the supernatant of the cell lysate of *E*. *coli* expressing fusion protein ACP-GFP treated by addition of the purified protease TEV; lane 4, target protein GFP purified by one-step Ni-chelating affinity chromatography.

**Fig 6:**
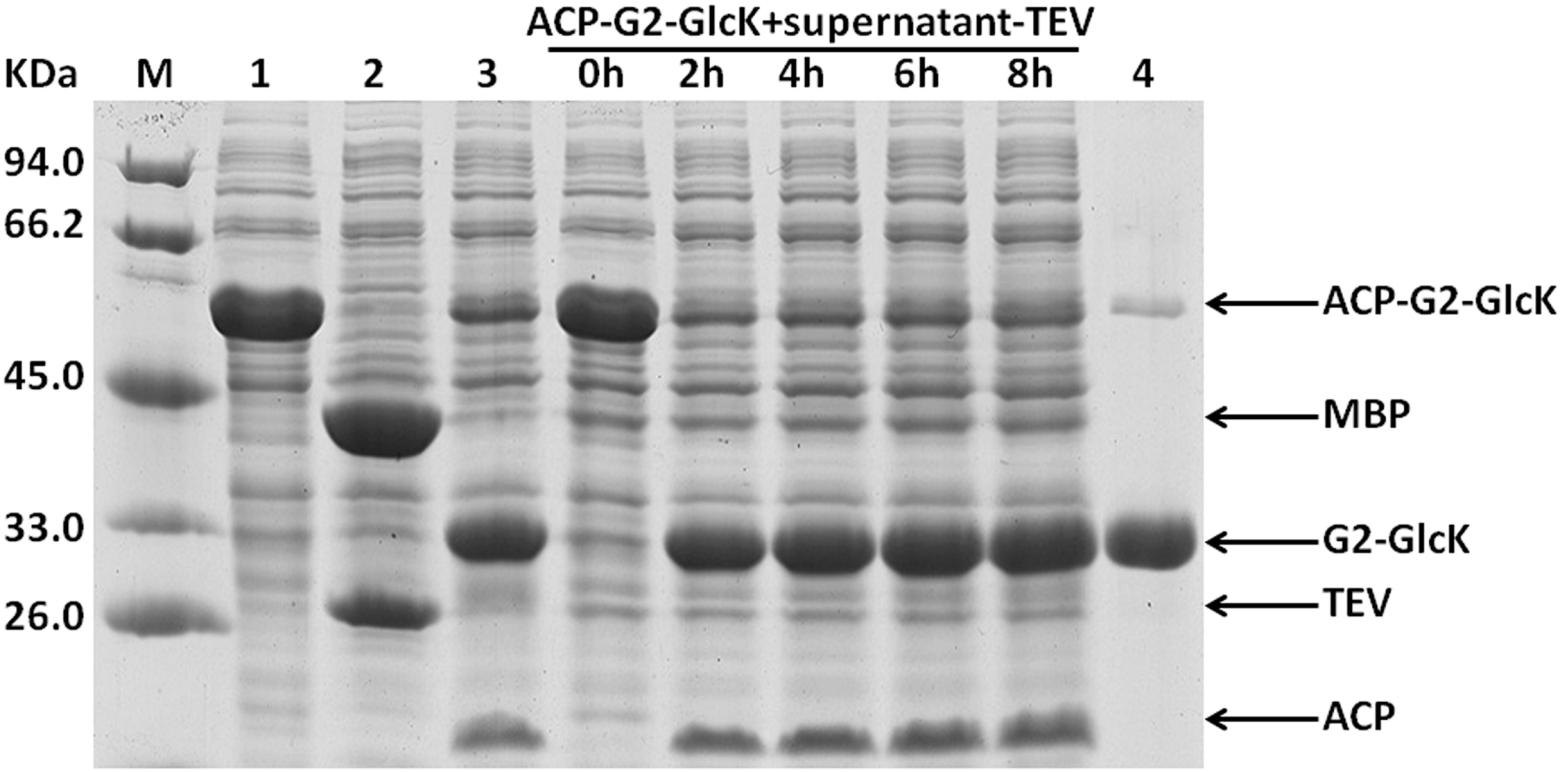
*In vitro* processing of the recombinant fusion protein ACP-G2-GlcK-Histag contained in *E*. *coli* cell lysate was performed by mixing it with the clarified cell lysate of *E*. *coli* over-expressing protease TEV (supernatant-TEV), and subsequently target protein GlcK was purified by one-step Ni-chelating chromatography. The supernatant of the cell lysate of *E*. *coli* expressing fusion protein ACP-G2-GlcK-Histag was mixed with supernatant-TEV at a volumetric ration of 10:1, and incubated at 25°C. Aliquots were removed for SDS-PAGE analysis after 0 h, 2 h, 4 h, 6 h and 8 h. lane 1, the supernatant of the cell lysate of *E*. *coli* expressing fusion protein ACP-G2-GlcK-Histag; lane 2, the supernatant of the cell lysate of *E*. *coli* expressing protease TEV by vector pET-28b-MBP-TEV-WHZ; lane 3, the supernatant of the cell lysate of *E*. *coli* expressing fusion protein ACP-G2-GlcK-Histag treated by addition of the purified protease TEV; lane 4, target protein GlcK purified by one-step Ni-chelating affinity chromatography.

**Fig 7:**
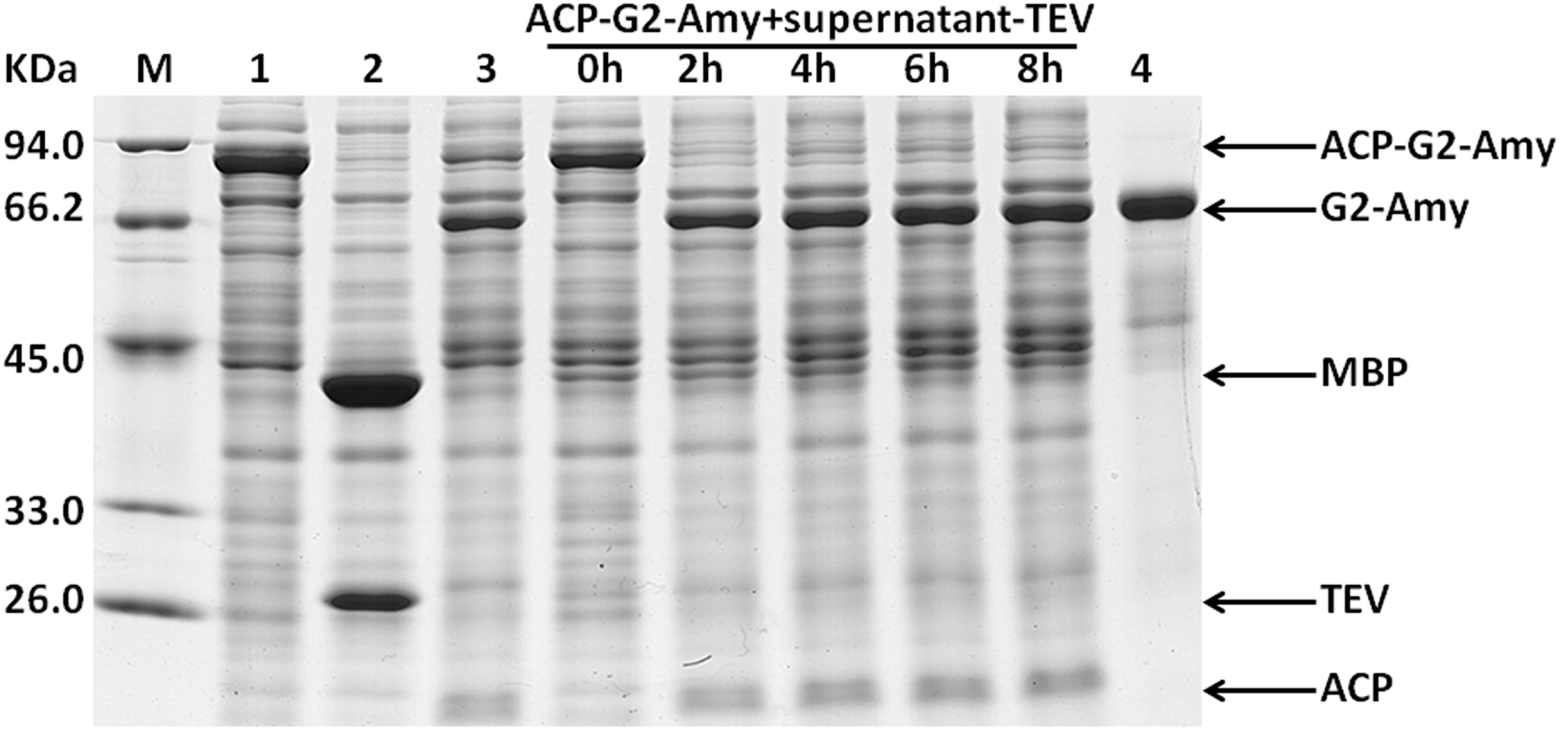
*In vitro* processing of the recombinant fusion protein ACP-G2-Amy-Histag contained in *E*. *coli* cell lysate was performed by mixing it with the clarified cell lysate of *E*. *coli* over-expressing protease TEV (supernatant-TEV), and subsequently target protein Amy was purified by one-step Ni-chelating chromatography. The supernatant of the cell lysate of *E*. *coli* expressing fusion protein ACP-G2-Amy-Histag was mixed with supernatant-TEV at a volumetric ration of 10:1 and incubated at 25°C. Aliquots were removed for SDS-PAGE analysis after 0 h, 2 h, 4 h, 6 h and 8 h. lane 1, the supernatant of the cell lysate of *E*. *coli* expressing fusion protein ACP-G2-Amy-Histag; lane 2, the supernatant of the cell lysate of *E*. *coli* expressing protease TEV by vector pET-28b-MBP-TEV-WHZ; lane 3, the supernatant of the cell lysate of *E*. *coli* expressing fusion protein ACP-G2-Amy-Histag treated by addition of the purified protease TEV; lane 4, target protein Amy purified by one-step Ni-chelating affinity chromatography.

A previous report has indicated that the MBP fusion proteins were cleaved right away when coexpressed with protease TEV in *E*. *coli*, while the resulting passenger proteins accumulated predominantly in an insoluble form [20]. Our data also show that the intracellular processing of fusion proteins by protease TEV coexpressed may bring about incomplete cleavage. In the fusion context, fusion tags may enhance the solubility of target proteins by promoting their proper folding via the spontaneous interaction between them. However, when the fusion proteins are coexpressed with protease TEV in *E*. *coli*, the fusion tag may be cleaved off the passenger protein soon after their being translated *in vivo*. The active roles of fusion tags are weakened. To settle this problem emerged probably under coexpression condition, we tried to develop another convenient procedure for purification of the target proteins expressed in fusion form. When expressed in *E*. *coli*, fusion protein MBP-TEV could be efficiently self-cleaved *in vivo*, leading to the accumulation of recombinant protease TEV in the supernatant of *E*. *coli* cell lysate [18,21]. We speculated that this kind of supernatant (Supernatant-TEV) could be used as an active reagent to process the fusion proteins *in vitro*. After being expressed in *E*. *coli*, fusion protein ACP-GFP-Histag contained in the supernatant of *E*. *coli* cell lysate was subjected to cleavage by mixing it with Supernatant-TEV. In the supernatant of recombinant *E*. *coli* cell lysate, if protease TEV could efficiently cleave fusion protein ACP-GFP-Histag, the soluble Histagged passenger protein GFP-Histag was produced. Consequently, target protein GFP-Histag could be further purified conveniently by one-step Ni-chelating affinity chromatography. The recovered supernatant containing fusion protein ACP-GFP-Histag was mixed with Supernatant-TEV at a volumetric ratio of 10:1, and then analyzed by SDS-PAGE to investigate the processing of ACP-GFP-Histag at different time intervals. As shown in Fig 5, our data clearly demonstrated that in the supernatant of *E*. *coli* cell lysate, protease TEV exhibited high activity, and the fusion protein ACP-GFP-Histag could be cleaved to completion within about 2 h. We also performed *in vitro* the processing of fusion protein ACP-G2-GlcK-Histag and ACP-G2-Amy-Histag contained in the supernatant of *E*. *coli* cell lysate by Supernatant-TEV. Similar results were obtained (Figs 6 and 7).

Our results clearly showed that the routine procedure for purification of recombinant protease TEV was not always necessitated, and the clarified cell lysate of *E*. *coli* expressing MBP-TEV could also be used as an effective reagent for removal of the fusion tag. Furthermore, enzymatic cleavage of recombinant fusion proteins in the supernatant of *E*. *coli* cell lysate made the resulting target proteins to be the only His-tagged proteins in the sample, which subsequently could be separated conveniently by just one-step Ni-chelating affinity chromatography (Figs 5, 6 and 7). Compared with the purification procedure described in Fig 2, the method presented here could also greatly simplify purification of the target proteins expressed in fusion form.

## Discussion

As one of the most widely used organisms for production of the foreign proteins of scientific, pharmaceutical, or industrial importance, *E*. *coli* expression system has several obstacles. Ready formation of inclusion bodies, low expression level and toxicity of recombinant proteins are generally the representatives of these difficulties. Fusion protein technology provides an alternative approach to solve these problems. However, removal of fusion tags from the fusion proteins is often necessitated since the fusion partners may potentially interfere with the proper structure and functions of passenger proteins [22]. Typically, for production of the pure target proteins expressed in fusion form, the fusion proteins are first separated by Ni-chelating affinity chromatography, and then dialyzed to the reaction buffer of endoproteases, cleaved *in vitro* by addition of the His-tagged endoproteases, and lastly subjected to the second subtractive Ni-chelating affinity chromatography to remove the fusion tags and endoproteases [12].

The procedure mentioned above is routinely utilized to purify the target proteins expressed in fusion form. Nevertheless, compared with one-step Ni-chelating affinity purification of target proteins from cell lysates, this procedure is time-costing, laborious and often brings about the lower productivity in light of the protein loss during dialysis and the second step of affinity chromatography. Otherwise, these purified endoproteases used for cleavage of the fusion proteins *in vitro* are very expensive. In large-scale production of the target proteins expressed in fusion form, the expense for use of the endoproteases constitutes a large portion of the total production cost [9]. The challenge facing us is how to develop a convenient procedure for production of the recombinant target proteins, which need to be expressed in fusion form.

When expressed in an unfused form in *E*. *coli*, the expression level of protein GlcK and Amy is much lower than expected for undetermined reasons. In this report, we find that fusion tag ACP can increase their expression level significantly when fused to their N-terminus. Fusion tags are widely utilized as solubility-enhancer. Nevertheless, our findings demonstrate that fusion tag genes can also be exploited to improve the expression of some heterogenous genes in *E*. *coli* and provide an alternative approach to increase the expression level of some target genes. In this case, to purify the target proteins conveniently, we try to coexpress protease TEV with the fusion proteins in *E*. *coli* for their site-specific cleavage *in vivo*. Protease TEV is a favorable candidate as an enzymatic reagent for removal of the fusion tags in light of its stringent sequence specificity and the established method for its expression in *E*. *coli* [23,24]. In addition, when expressed in fusion form in *E*. *coli*, the recombinant protease TEV can exhibit high activity *in vivo*, indicated by efficient self-cleavage of the fusion protein MBP-TEV [18,24,25]. As expected, under coexpression condition, fusion protein ACP-G2-GlcK-Histag and ACP-G2-Amy-Histag can be efficiently cleaved *in vivo* by the protease TEV coexpressed, leading to generation of the soluble His-tagged target proteins, which subsequently can be conveniently purified by one-step Ni-chelating affinity chromatography. Generally, for purification of passenger proteins (target proteins) by Ni-chelating affinity chromatography, a His-tag is often appended at its C-terminus. Nevertheless, the His-tag can also be inserted between the protease TEV recognition site and the N-terminus of passenger protein, if necessary. The location of a His-tag at the C-terminus or N-terminus of passenger proteins does not affect their expression level and subsequent cleavage *in vivo* (data not shown). The strategy presented here not only can exploit the beneficial effect of fusion tags on the expression level of their passenger proteins, and also eliminate the processing of fusion proteins with the purified site-specific proteases *in vitro* and subsequent removal of the fusion tags as well as these endoproteases. Consequently, the procedure for purification of the target proteins expressed in fusion form is greatly simplified.

As a fusion tag, ACP can greatly increase the expression level of passenger protein Amy and GlcK in *E*. *coli*. However, the mechanism of its active roles in improving their expression level is unclear. We do not know whether the improvement in expression level of its passenger proteins is correlated with their correct folding promoted by the fusion partner ACP. In fusion form, the active roles of fusion tag ACP may be performed by its spontaneous interaction with passenger proteins. Whereas under coexpression condition, processing of fusion proteins by protease TEV is in parallel with folding of passenger proteins *in vivo*, resulting in attenuation of the positive action of fusion tag ACP. In fact, passenger protein GlcK-Histag and Amy-Histag cleaved off the fusion tag can accumulate predominantly in a soluble form *in vivo* and retain their natural activity, implying that protein GlcK and Amy may be ready to fold correctly even without help of the fusion tag ACP in *E*. *coli*.

In many cases, fusion tags function mainly as solubility-enhancer. Previous report has noted that intracellular processing of fusion proteins by the protease TEV coexpressed resulted in aggregation of passenger proteins [20,26]. The solubility of some passenger proteins is influenced significantly by the duration of their association with fusion tags, especially for the proteins tending to form aggregates when overexpressed in *E*. *coli*. In this case, coexpression of fusion proteins with site-specific proteases probably brings about production of insoluble passenger proteins. A challenge faces us that how to develop a procedure, which can take into account both the enhanced soluble expression of target proteins and its subsequent convenient purification. In our laboratory, it is found that the supernatant of the cell lysate of *E*. *coli* expressing protease TEV (supernatant-TEV) can preserve high cleavage activity for over 1 year at -70°C (data not shown). It is reasonable to speculate that the supernatant-TEV can be utilized as an active reagent to cleave the fusion proteins *in vitro*. Our results presented here showed that it took only about 2 h for the fusion proteins contained in the supernatant of *E*. *coli* cell lysate to be cleaved completely just by mixing them with the supernatant-TEV at a volumetric ratio of 10:1, suggesting that the cleavage efficiency is rather high. In this scheme, the His-tag for affinity chromatography can be appended at the C-terminus of a passenger protein or inserted between the protease TEV recognition site and the N-terminus of a passenger protein as needed, while recombinant protease TEV is not His-tagged. After being processed *in vitro*, a fusion protein will be cleaved into the fusion tag and the soluble His-tagged passenger protein (target protein), which subsequently can be purified conveniently by just one-step Ni-chelating affinity chromatography. Thus, utilization of the supernatant-TEV to cleave fusion proteins not only makes purification of passenger proteins greatly simplified, and the solubility of cleaved passenger proteins can be protected. This strategy can also eliminate need for large amounts of the pure protease TEV at a later stage. It is interesting to note that passenger protein GFP cleaved off the fusion tag ACP *in vitro* can maintain its soluble state, while it tends to form aggregates after being cleaved off the same fusion tag *in vivo*. Fusion tag ACP may be cleaved off soon after the fusion protein ACP-ACP-Histag is translated under coexpression condition. The duration of its association with fusion tag ACP may greatly influence its solubility.

**Fig 8:**
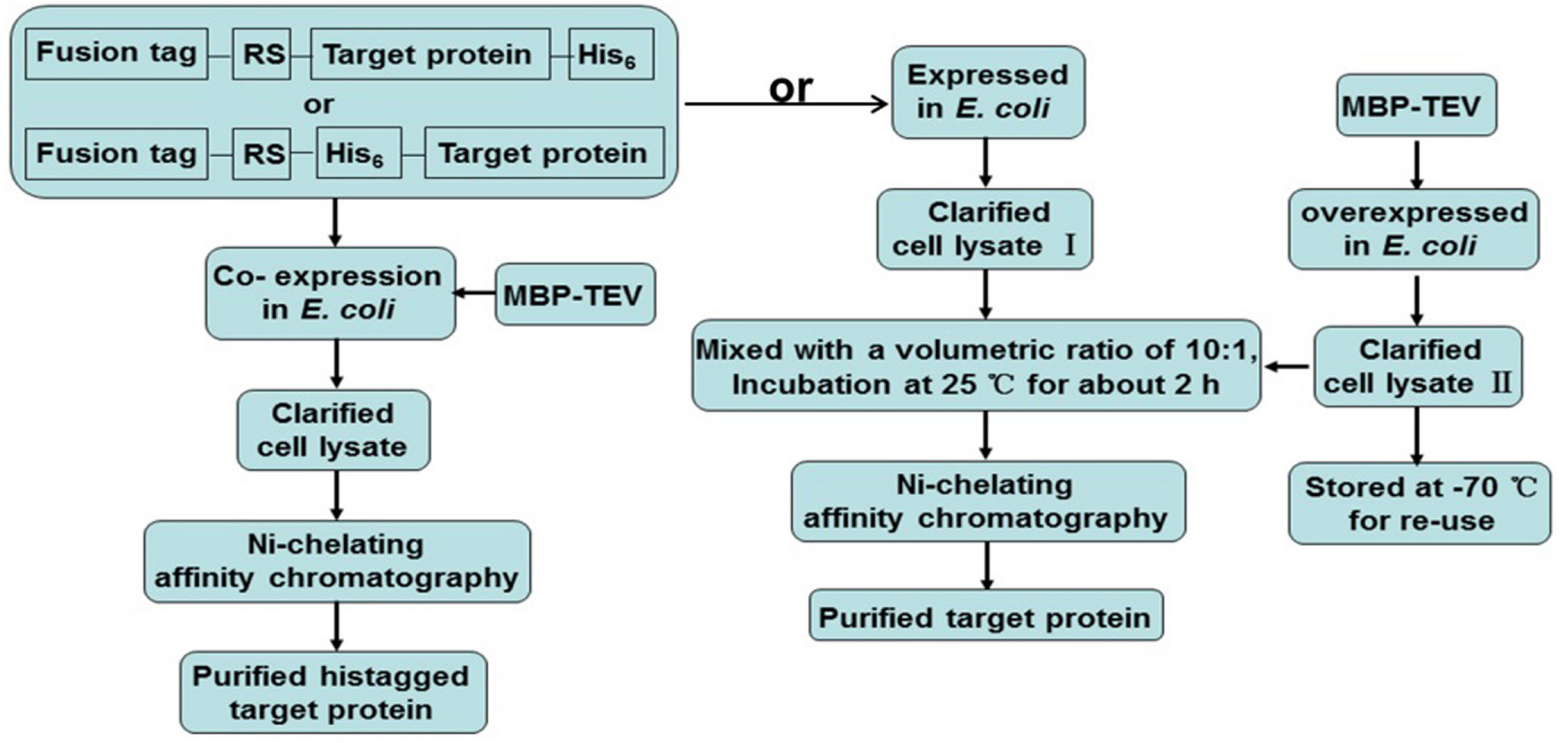
The protocol presented for purification of the target proteins, which need to be expressed in fusion form. The passenger proteins that either accumulate dominantly in the soluble form after being cleaved off the fusion tags by the site-specific protease TEV co-expressed *in vivo* or are ready to form aggregates when processed intracellularly, can be convenient purified by one-step Ni-chelating affinity chromatography; RS: protease TEV recognition site.

Overall, in this report we present two approaches for convenient production of the recombinant target proteins, which need to be expressed in fusion form to increase their soluble expression level. They are outlined in Fig 8. These approaches not only eliminate purification of the site-specific protease TEV, and greatly simplify the procedure for purification of target proteins.

## Materials and Methods

### Bacterial strains and vectors

*E*. *coli* strain BL21 (DE3), based on T7 expression systems, was selected for expression of recombinant proteins. Three expression vectors were used: the pET-28b (Novagen), pSU18 and pBAD34. The pET-28b allows overexpression of recombinant proteins. The target genes cloned in pSU18 and pBAD34 could be induced by isopropyl β-D-1-thiogalactopyranoside (IPTG) and arabinose respectively for their expression at physiological level [27,28]. The pSU18, pBAD34 and their derivative vectors are all compatible with pET serial vectors.

### Molecular cloning and construction of expression vectors

Acyl carrier protein (ACP) gene (accession number, Gene ID: 944805) was first amplified from *E*. *coli* (strain MG1655) chromosome with primer-1 (CATCATCATCATCACGGTGGTAGCACTATCGAAGAACGCG) and primer-2 (TCGAATTCAGCACCACCACCACCAGAAGCCGCCTGGTGGCCGTTGATG), and then the amplicon was used as the template for the second amplification of fusion ACP gene with primer-3 (ATACCATGGGCCATCATCATCATCATCACGGTG) and primer-2, which encoded protein ACP with a Histag at its N-terminus and a flexible peptide (ASGGGGA) at its C-terminus. The PCR product was digested with *Nco* I and *Bam*H I, and then inserted into vector pET-28b, generating vector pET-28b-Histag-ACP. Glucokinase (GlcK) gene (accession number, Gene ID: 938206) was amplified from *Bacillus subtilis* (strain 168) chromosome with primer-4 (CATCATCATCATCACGGTGGTGACGAGATATGGTTTGCG) and primer-5 (AGCAAGCTTAACAATTTTGATGTTTCAG), and then the amplicon was used as the template for the second amplification of GlcK gene with primer-3 and primer-5. The PCR product was digested with *Nco* I and *Hin*d III, and inserted into vector pET-28b, generating vector pET-28b-Histag-GlcK. Similarly, alpha-Amylase (Amy) gene (accession number, Gene ID: 938356) was amplified from *Bacillus subtilis* (strain 168) chromosome first with primer-6 (CATCATCATCATCACGGTGGTCTTACAGCACCGTCGATC) and primer-7 (ACTAAGCTTAATGGGGAAGAGAACCGCTTAAG), then with primer-3 and primer-7. The PCR product was digested with *Nco* I and *Hin*d III, and inserted into vector pET-28b, generating vector pET-28b-Histag-Amy.

For fusion expression of GlcK and Amy, the protease TEV recognition site (ENLYFQ) was appended at the N-terminus of GlcK and Amy, respectively. Fusion GlcK gene was amplified with primer-8 (TCCGAATTCGAAAACCTGTACTTCCAGGACGAGATATGGTTTGCG) and primer-5 using vector pET-28b-Histag-GlcK as template, the resulting amplicon was digested with *Eco*R I and *Hin*d III, and then inserted into vector pET-28b-Histag-ACP, generating vector pET-28b-Histag-ACP-GlcK for overexpression of the fusion protein Histag-ACP-GlcK in *E*. *coli*. Meanwhile, another fusion GlcK gene was also amplified with primer-9 (TCCGAATTCGAAAACCTGTACTTCCAGGGTGGTGACGAGATATGGTTTGCG) and primer-5, the resulting amplicon was digested with *Eco*R I and *Hin*d III, and inserted into vector pET-28b-Histag-ACP, generating vector pET-28b-Histag-ACP-G_2_-GlcK for overexpression of the fusion protein Histag-ACP-G_2_-GlcK in *E*. *coli*. Two consecutive glycine residues were inserted between the TEV recognition site and protein GlcK in the fusion protein Histag-ACP-GlcK, bringing about production of its counterpart Histag-ACP-G_2_-GlcK. Similarly, fusion Amy gene was amplified with primer-10 (TCCGAATTCGAAAACCTGTACTTCCAGCTTACAGCACCGTCGATC) and primer-7, then the resulting amplicon was digested with *Eco*R I and *Hin*d III, and inserted into plasmid pET-28b-Histag-ACP to construct the vector pET-28b-Histag-ACP-Amy for overexpression of the fusion protein Histag-ACP-Amy; with primer-11 (TCCGAATTCGAAAACCTGTACTTCCAGGGTGGTCTTACAGCACCGTCGATC) and primer-7 to construct the vector pET-28b-Histag-ACP-G_2_-Amy for overexpression of the fusion protein Histag-ACP-G_2_-Amy. We also constructed vector pET-28b-ACP-G_2_-GlcK-Histag and pET-28b-ACP-G_2_-Amy-Histag for overexpression of the fusion protein ACP-G_2_-GlcK-Histag and ACP-G_2_-Amy-Histag respectively, with a Histag at their C-terminus. The fusion gene *ACP-G*_*2*_ *-GlcK-Histag* was first amplified with primer-12 (ATACCATGGGCAGCACTATCGAAGAACGCG) and primer-13 (ATGATGATGATGGCCACCACAATTTTGATGTTTCAGCC), then with primer-12 and primer-14 (TGGAAGCTTAGTGATGATGATGATGATGGCCACC) using vector pET-28b-Histag-ACP-G_2_-GlcK as template. The resulting amplicon was digested with *Nco* I and *Hin*d III, and then inserted into vector pET-28b, generating vector pET-28b-ACP-G_2_-GlcK-Histag for overexpression of the fusion protein ACP-G_2_-GlcK-Histag (ACP-G_2_-GlcK with a Histag at its C-terminus) in *E*. *coli*. Similarly, the fusion gene *ACP-G*_*2*_ *-Amy-Histag* was first amplified with primer-12 and primer-15 (ATGATGATGATGGCCACCATGGGGAAGAGAACCGCTTAAG), then with primer-12 and primer-14 using vector pET-28b-Histag-ACP-G_2_-Amy as template. The resulting amplicon was digested with *Nco* I and *Hin*d III, and then inserted into vector pET-28b, generating vector pET-28b-ACP-G_2_-Amy-Histag for overexpression of the fusion protein ACP-G_2_-Amy-Histag (ACP-G_2_-Amy with a Histag at its C-terminus) in *E*. *coli*. For fusion expression of protein GFP, fusion GFP gene was amplified with primer-16 (TCCGAATTCGAAAACCTGTACTTCCAGGTGAGCAAGGGCGAGGAG) and primer-17 (TCAAGCTTAGTGATGATGATGATGATGCTTGTACAGCTCGTCCATG) using the vector pET-30a-GFP constructed previously as template. The resulting amplicon was digested with *Eco*R I and *Hin*d III, and then inserted into the vector pET-28b-ACP constructed previously, generating vector pET-28b-ACP-GFP-Histag for overexpression of the fusion protein ACP-GFP-Histag (ACP-GFP with a Histag at its C-terminus and a protease TEV recognition site between ACP and GFP) in *E*. *coli*.

The vector pET30a-MBP-TEV was constructed previously for overexpression of the protease TEV with a Histag at its N-terminus [18]. Protease TEV gene was amplified with primer-18 (TCCGAATTCGAAAACCTGTATTTCCAGGGCGGTGAATCTCTGTTCAAAG) and primer-19 (TGCTAGTTATTGCTCAGCGG) using vector pET30a-MBP-TEV as template. The resulting amplicon was digested with *Eco*R I and *Hin*d III, and then inserted into vector pET-28b-MBP constructed previously, generating vector pET-28b-MBP-TEV-WHZ for overexpression of the de-tagged protease TEV. To achieve coexpression of protease TEV with the fusion proteins in *E*. *coli*, we also constructed the vector pBAD34-MBP-TEV-WHZ and pSU18-MBP-TEV-WHZ for expression of the de-tagged protease TEV. Fusion gene *MBP-TEV* was amplified with primer-20 (TAATACGACTCACTATAGGG) and primer-19 using vector pET-28b-MBP-TEV-WHZ as template. The resulting amplicon was digested with *Nco* I and *Hin*d III, and then inserted into pBAD34, generating vector pBAD34-MBP-TEV-WHZ; digested with *Xba*l I and *Hin*d III, and then inserted into pSU18, generating vector pSU18-MBP-TEV-WHZ. All vectors constructed were confirmed by sequencing. Characterization of these vectors was outlined in Table 1.

**Table 1:** Characterization of vectors constructed.

Vectors	Characterization
pET-28b-histag-ACP	Encoding protein ACP with a his_6_-tag at its N-terminus, a flexible peptide (ASGGGGA) at its C-terminus.
pET-28b-histag-ACP-GlcK	For expression of the fusion protein ACP-GlcK with a his_6_-tag at its N-terminus, in which the protease TEV recognition site (ENLYFQ) is inserted between ACP and GlcK.
pET-28b-histag-ACP-G2-GlcK	For expression of the fusion protein ACP-GlcK with a his_6_-tag at its N-terminus, in which two consecutive glycine residues are inserted between the TEV recognition site (ENLYFQ) and GlcK.
pET-28b-ACP-G2-GlcK-histag	For expression of the fusion protein ACP-GlcK with a his_6_-tag at its C-terminus, in which two consecutive glycine residues are inserted between the TEV recognition site (ENLYFQ) and GlcK.
pET-28b-histag-ACP-Amy	For expression of the fusion protein ACP-Amy with a his_6_-tag at its N-terminus, in which the protease TEV recognition site (ENLYFQ) is inserted between ACP and Amy.
pET-28b-histag-ACP-G2-Amy	For expression of the fusion protein ACP-Amy with a his_6_-tag at its N-terminus, in which two consecutive glycine residues are inserted between the TEV recognition site (ENLYFQ) and Amy.
pET-28b-ACP-G2-Amy-histag	For expression of the fusion protein ACP-Amy with a his_6_-tag at its C-terminus, in which two consecutive glycine residues are inserted between the TEV recognition site (ENLYFQ) and Amy.
pET-28b-MBP-TEV-WHZ	For overexpression of the detagged protease TEV
pBAD34-MBP-TEV-WHZ	For coexpression of the protease TEV with fusion proteins *in vivo*; the expression of protease TEV is induced by arabinose.
pSU18-MBP-TEV-WHZ	For coexpression of the protease TEV with fusion proteins *in vivo*; the expression of protease TEV is induced by IPTG.

### Growth media

For the gene cloning and protein expression experiments, *E*. *coli* cells were cultivated in LB medium. The culture medium prepared was supplemented with 50 μg/ml of kanamycin for the *E*. *coli* cells encompassing pET-28b and its derivative vectors; 100 μg/ml of ampicillin for pBAD34 derivative vectors; 30 μg/ml of chloramphenicol for pSU18 derivative vectors.

### Recombinant protein expression

Six milliliters of LB medium, supplemented with antibiotics as needed, was inoculated by a single colony of *E*. *coli* BL21 (DE3) cells transformed with vectors constructed, and cultured overnight at 37°C under agitation (200 rpm). 100 μl of overnight culture was then added to 10 ml of fresh LB medium, which was allowed to grow until they reached the mid-log phase (with an optical density of around 0.6–0.8 at 600 nm). The expression of recombinant proteins was induced by addition of 0.2% arabinose for the *E*. *coli* cells encompassing pBAD34-MBP-TEV-WHZ; 0.4 mM of IPTG for the *E*. *coli* cells encompassing pSU18-MBP-TEV-WHZ or pET derivative vectors. After that, the cultures were incubated for 12 h at 20°C under agitation (150 rpm).

10 ml of cultures was harvested by centrifugation at 1500g for 10 min, resuspended in 4 mL lysis buffer (50 mM NaH_2_PO_4_, 0.3 M NaCl, 20 mM imidazole, pH 8.0), and then lysed by sonication on ice. After sonication, the lysate was centrifuged at 13,500g for 20 min. The supernatant was then recovered, and the insoluble fraction was taken as inclusion bodies and dissolved in the phosphate buffer (50 mM NaH_2_PO_4_, pH 7.0, 8 M urea). The supernatants and inclusion bodies were further analyzed by 12% SDS-PAGE.

### Affinity purification of recombinant proteins

The supernatants of *E*. *coli* cell lysates containing recombinant proteins were directly loaded onto a Ni–Sepharose affinity column (GE Healthcare). The loaded column was washed three times with washing buffer (50 mM NaH_2_PO_4_, 300 mM NaCl, 40 mM imidazole, pH 8.0). After that, the recombinant proteins was eluted with elution buffer (50 mM NaH_2_PO_4_, 300 mM NaCl, 250 mM imidazole, pH 8.0), and evaluated by 12% SDS-PAGE.

After that, purified fusion protein Histag-ACP-G_2_-GlcK (or Histag-ACP-G_2_-Amy) contained in the elution buffer was subjected to cleavage *in vitro* by mixing it with purified His-tagged protease TEV according to the procedure reported previously [18]. Then, the mixture was dialyzed to lysis buffer (50 mM NaH_2_PO_4_, 300 mM NaCl, 20 mM imidazole, pH 8.0), and subjected to the second subtractive Ni-chelating affinity chromatography to remove His-tagged ACP and protease TEV. Target protein GlcK (or Amy) was recovered in the flow-through sample, and analyzed by 12% SDS-PAGE.

### Assay of recombinant Amy and GlcK activity

Recombinant α-Amylase activity was assayed based on the 3, 5-dinitrosalicylic acid method. The reaction mixture (200 ml) consisted of 0.5% soluble starch in 20mM sodium acetate buffer (pH 5.5) and the enzyme. The reaction was stopped after 10 min of incubation at 55°C by the addition of 3, 5-dinitrosalicylic acid reagent (200 ml). The reagent was prepared by mixing 0.4 M NaOH, 22 mM 3, 5-dinitrosalicylic acid, and 1.1 M potassium sodium-tartrate tetrahydrate. One unit of enzyme activity was defined as the amount of enzyme that released 1 μmol of reducing sugar as glucose per min under the assay conditions described above.

During measurement of Glucokinase activity, the formation of glucose 6-phosphate (G6P) was coupled to the reduction of NADP through glucose-6-phosphate dehydrogenase (G6PD). The reaction mixture contained (in 2 ml): 50 mM triethanolamine/HCl, pH 7.4, 10 mM MgC1, 0.5 mM NADP, 1 mM ATP, 1.4 units G6PD and up to 50 μl of recombinant GlcK. The solution was divided between two cuvettes and, after monitoring the A340 for 1 min, the reaction was started by addition of glucose (10 mM) to the sample cuvette. One unit of GlcK activity was defined as the amount of enzyme catalyzing conversion of 1 μmol of Glucose in 1 min at room temperature.

### Processing of the fusion proteins contained in the clarified *E*. *coli* cell lysate

After being induced by IPTG, *E*. *coli* cells encompassing pET28b-MBP-TEV-WHZ were lysed by sonication. The supernatant of cell lysate was recovered (Supernatant-TEV), and utilized as the reagent for processing of the fusion proteins *in vitro*. The fusion protein ACP-GFP-Histag (ACP-G_2_-Amy-Histag or ACP-G_2_-GlcK-Histag) was subjected to cleavage *in vitro* by mixing them with Supernatant-TEV at a volumetric ration of 10:1. The digest reaction was performed at 25°C for 2 h, 4 h, 6h, or 8 h respectively. Aliquots were removed for SDS-PAGE analysis.
